# Needle-based gastrocnemius lengthening: a novel ultrasound-guided noninvasive technique

**DOI:** 10.1186/s13018-022-03318-8

**Published:** 2022-09-29

**Authors:** Álvaro Iborra Marcos, Manuel Villanueva Martínez, Homid Fahandezh-Saddi Díaz

**Affiliations:** 1 Unit for Ultrasound-guided Surgery, Hospital Beata María Ana, Madrid, Spain; 2 Avanfi Institute, Calle Donoso Cortes, 80, 28015 Madrid, Spain; 3 Faculty of Health Sciences, Department of Podiatry, University of La Salle, Barcelona, Spain

**Keywords:** Equinus foot, Distal gastrocnemius recession, Ultrasound-guided surgery, Gastrocnemius tendon lengthening

## Abstract

**Background:**

Gastrocnemius tendon lengthening is performed to treat numerous conditions of the foot and ankle. Gastrocnemius shortening has been associated with more than 30 lower limb disorders, including plantar heel pain/plantar fasciitis, Achilles tendinosis, equinus foot, adult flat foot deformity, and metatarsalgia. Ultrasound-guided ultraminimally invasive lengthening of the gastrocnemius is a step forward in this type of surgery. It can be performed in both legs simultaneously without ischemia using only local anesthesia plus sedation and without the need for a cast or immobilization. The truly novel advantage of the procedure is that it can be performed in the office, without specific surgical instruments. The aim of our research was to prove the effectiveness and safety of a new closed needle-based ultrasound-guided surgical procedure for lengthening the gastrocnemius tendon.

**Methods and results:**

We performed ultrasound-guided gastrocnemius tendon lengthening using a needle in eight fresh frozen specimens (3 left and 5 right). None of the specimens had been affected by disease or undergone previous surgery that could have affected the surgical technique. We used a linear transducer with an 8- to 17-MHz linear transducer and the beveled tip of an Abbocath as a surgical blade to perform the lengthening procedure. The gastrocnemius Achilles tendon recession was entirely transected in all eight specimens, with no damage to the sural nerve or vessels. The improvement in dorsal flexion was 15°.

**Conclusion:**

Needle-based ultrasound-guided gastrocnemius tendon lengthening is safe, since the surgeon can see all structures clearly, thus minimizing the risk of damage. The absence of a wound obviates the need for stitches, and recovery seems to be faster. The procedure can be performed in a specialist's office, as no specific surgical instruments are required. This technique could be a valid option for gastrocnemius lengthening and may even be less traumatic than using a hook-knife, as in our previous description.

## Introduction

Gastrocnemius contracture has been described as “the most profound causal agent in foot pathomechanics,” and “the primary causal agent in a significant proportion of foot pathology” [1]. 

Equinus is defined as ankle dorsiflexion < 10° with the knee extended [2–11].

Barouk et al. [12] described the Silfverskiöld maneuver as the appropriate technique for differentiating gastrocnemius equinus from gastrocnemius-soleus equinus. DiGiovanni et al. recommended a definition based on their study: ankle joint dorsiflexion < 5° with the knee extended for gastrocnemius equinus and < 10° with the knee flexed for gastrocnemius-soleus equinus is considered as abnormal [13].

Isolated gastrocnemius contracture is believed to lead to numerous pathological conditions of the foot and ankle and has been associated with more than 30 lower limb disorders, including plantar heel pain/plantar fasciitis, Achilles tendonitis/tendinosis, adult flat foot deformity, metatarsalgia, hallux abductus valgus, hammer toe/claw toe, hallux limitus/rigidus, and forefoot nerve entrapment [1, 8, 14].

In children, the deformity has been associated with clubfoot, spasticity, and cerebral palsy [2].

Therefore, gastrocnemius recession, either alone or in combination with other techniques, has many well-documented indications.

It is considered indicated in adults with dorsiflexion < 10° with the knee extended [15, 16].

Gastrocnemius muscle lengthening was first described in 1913 by Vulpius and Stoeffel [17] and subsequently by Silfverskiöld and other authors, who described surgical approaches at different anatomical levels of the gastrocnemius complex [18–21].

Gastrocnemius recession can be performed proximally or distally as open surgery, as an endoscopic procedure, or as ultrasound-guided surgery [11, 22]. While many open techniques have been described, these are associated with poor cosmetic results, neurovascular compromise, and wound dehiscence. Complications can lead to patient dissatisfaction [11, 22–27].

Endoscopic gastrocnemius recession appears to be less invasive and may have advantages over open procedures in terms of skin damage, smaller incisions, shorter recovery times, fewer complications, and reduced morbidity [28], although sural nerve injury and the need for limb exsanguination may be limitations [29].

Open and endoscopic procedures require epidural anesthesia, lower limb ischemia, and sutures.

Various ultrasound-guided surgical techniques for gastrocnemius resection have been described [11, 22, 30]. In 2016 Villanueva et al [11], first described ultrasound-guided gastrocnemius resection, which consists of sectioning the gastrocnemius tendon through anatomical level II, as in the "Strayer technique". In 2018, the same authors described proximal resection of the medial head of the calf muscle [11, 22].

Ultrasound-guided gastrocnemius recession is performed without ischemia and with local anesthesia plus sedation and does not require stitches. The potential benefits include shorter recovery time, fewer complications, reduced morbidity, and the possibility of performing bilateral procedures alone or in combination with other ultrasound-guided surgical techniques on an outpatient basis [11, 22].

Ultrasound-guided gastrocnemius resection appears to be the most advantageous of currently used techniques. The incision is 1 mm, and ischemia is unnecessary, thus minimizing the complications typical of open and endoscopic surgery. In addition, since it enables direct and continuous visualization of all structures, the possibility of neurovascular damage is reduced [11, 22].

Complications were minimal in our series, mainly hematomas that were reabsorbed at 3–4 weeks. Proximal recession was necessary in only one patient, who experienced slight cutaneous dysesthesia in the proximal third of the calf [28].

We previously applied aponeurotomy for Dupuytren’s disease and needle-based plantar fasciotomy. Ultrasound-guided release of the carpal tunnel was described by McShane et al. 10 years ago. To our knowledge, needle-based gastrocnemius lengthening has not been previously described. We believe that this viable and reproducible surgical procedure warrants further consideration [31–33].

The objective of this study was to evaluate the safety and efficacy of ultrasound-guided recession of the gastrocnemius tendon at level II using an Abbocath.

We evaluated the range of dorsiflexion before and after the procedure and assessed possible complications, including neurovascular injuries.

## Material and methods

This prospective cadaver study was performed in accordance with the principles of the 1964 Declaration of Helsinki (2013 revision). The specimens were provided by the Department of Anatomy of University Lasalle (Madrid, Spain). We used eight fresh anatomic specimens (3 left and 5 right) including the knee, calf, ankle, and foot.

All procedures were performed by two surgeons with 10 years’ experience in ultrasound-guided surgery. A maximum volume of 30 mL of saline solution was injected to create a working space for the needle by dissection of the tissues, mainly the sural nerve and vein.

It was important for the cadaver specimens not to touch the walls of the storage box to prevent damage to the skin and, therefore, alterations in echogenicity, which could have slowed the learning curve.

**Fig. 1:**
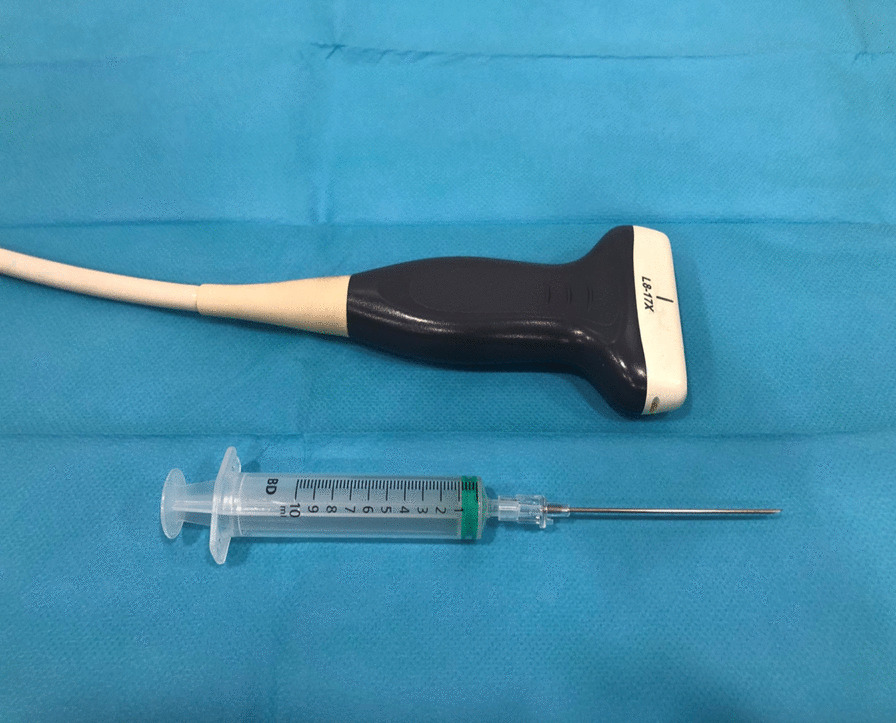
Instrument set: L8-17X multifrequency linear transducer (Alpinion Medical Systems, Bothell, WA, USA) and Abbocath 16 G needle

The basis of our technique has been reported [33] elsewhere. The ultrasound device was an E-CUBE 15 with an L8-17X multifrequency linear transducer (Alpinion Medical Systems, Bothell, WA, USA) and the Needle Vision Plus™ software package (Alpinion Medical Systems, Bothell, WA, USA) (Fig. 1).

All patients underwent ultrasound-guided gastrocnemius recession in level II using an Abbocath 16 G needle.

All procedures were performed by injecting a maximum volume of 30 mL of saline solution to create a working space for the needle and therefore enable dissection of the tissues between the fascia and the gastrocnemius tendon and beneath the sural nerve and vein.

### Surgical technique

The specimen was placed prone on the operating table. Ultrasound images in the transverse plane were used to delineate the anatomic boundaries of the gastrocnemius Achilles tendon, soleus muscle, and sural nerve and vein.

The point for recession of the tendon was chosen within level II, usually 2 cm distal to the distal end of the medial gastrocnemius belly, although, in theory, any point within level II could be chosen without the risk of uncontrolled lengthening or of creating unstable gait. We used ultrasound to identify the tendon and the sural nerve and vein.

**Fig. 2:**
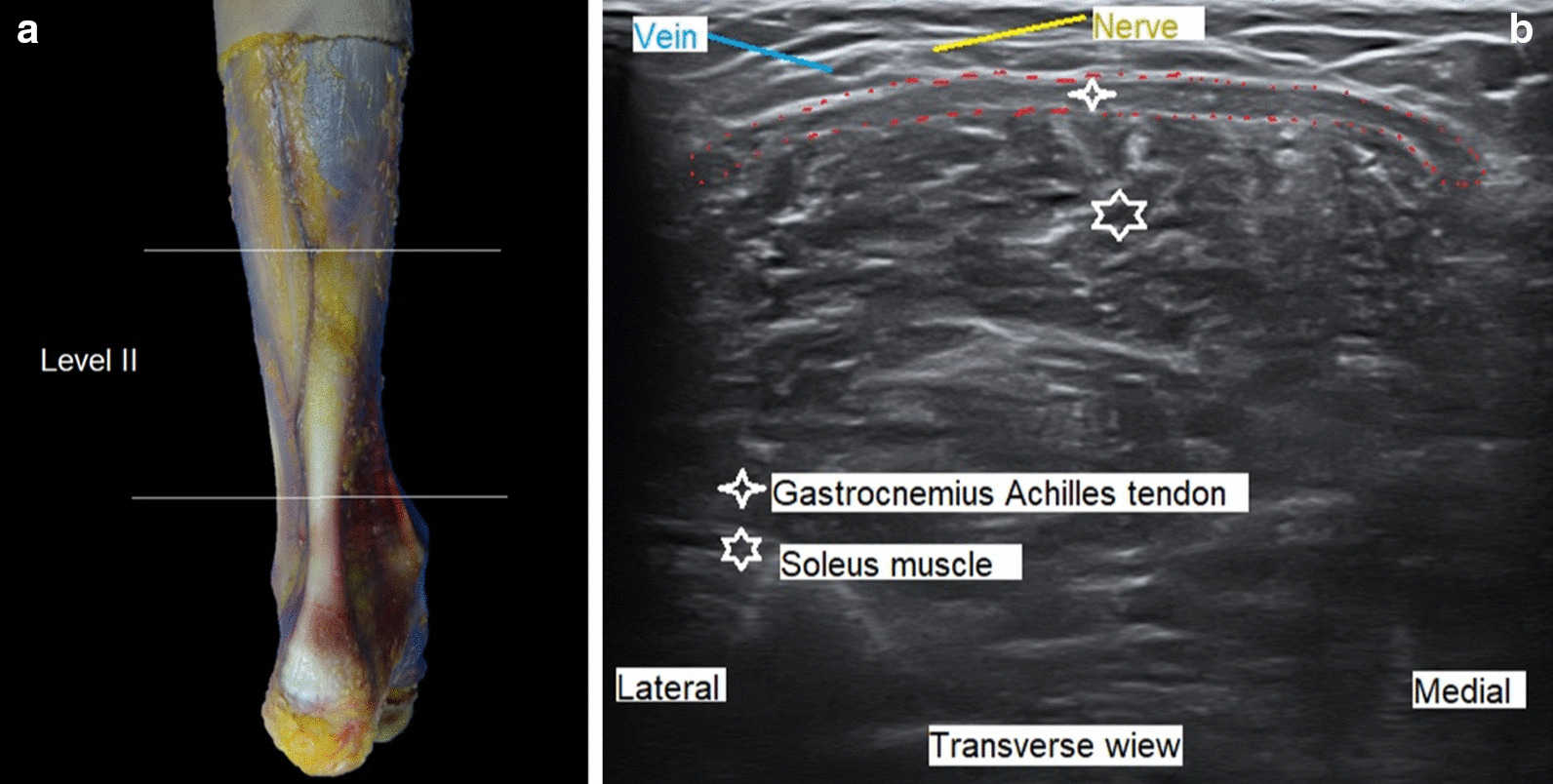
**a** Delimitation of the gastrocnemius Achilles tendon at level II. **b** Ultrasound with transducer in the short or transverse axis, identification of the nerve and sural vessels

With the transducer in the short or transverse axis, the nerve and sural vessels are always visible (Fig. 2a, b).

**Fig. 3:**
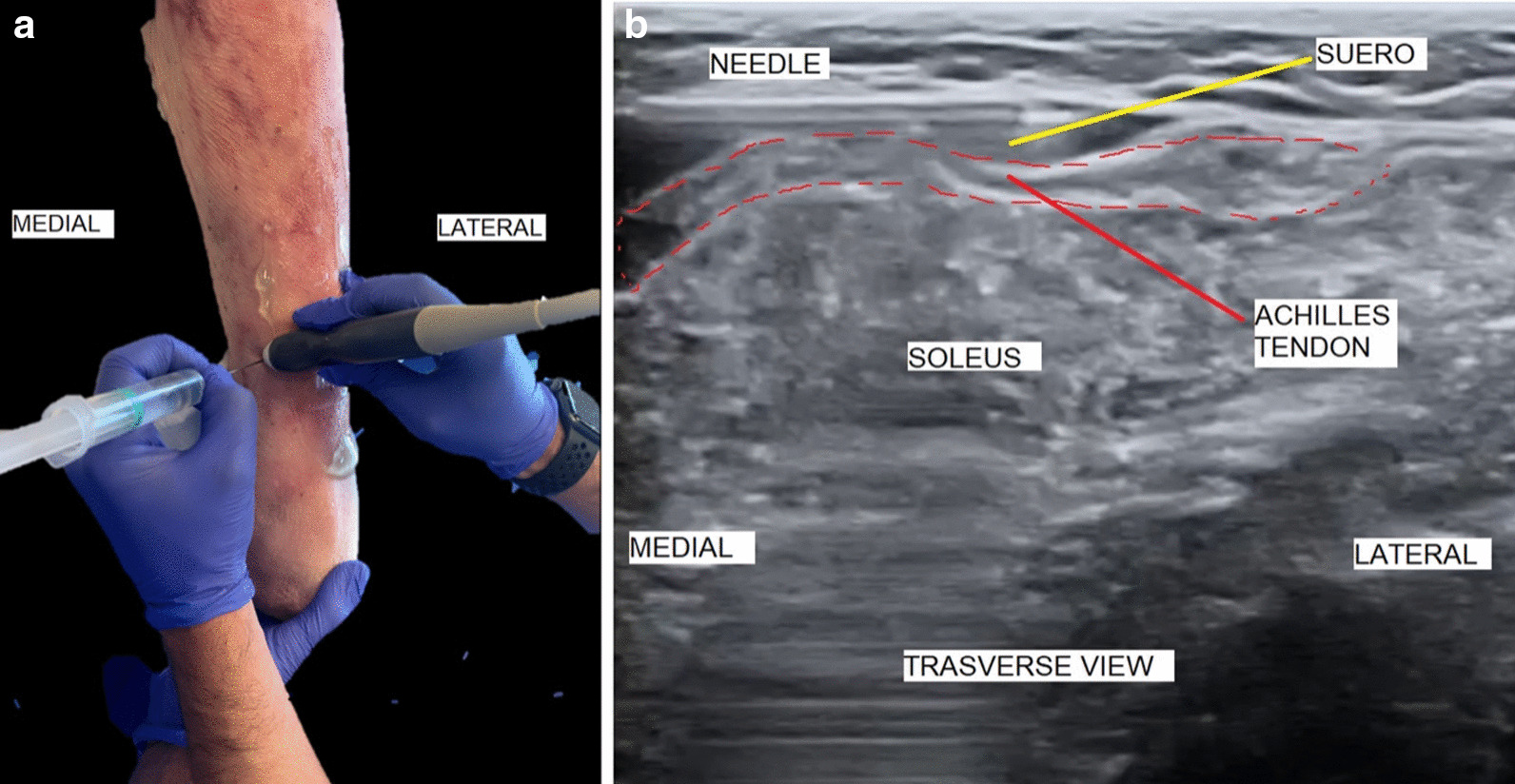
**a**, **b** Injecting serum between the nerve and the gastrocnemius Achilles tendon

At the chosen entry point, we inserted the 16 G needle from medial to lateral, with both the needle and the transducer transverse to the gastrocnemius tendon. The needle was then placed between the gastrocnemius tendon and the soleus muscle and fascia. At the desired level, we injected serum underneath the fascia to create a working space between the nerve, the underlying fascia, the gastrocnemius tendon, and the soleus muscle. The needle was clearly visible at all times (Fig. 3a, b).

With the ultrasound in the transverse position, the needle was used to repeatedly pierce the gastrocnemius Achilles tendon from medial to lateral. After the initial piercings, we began to move the needle using a windscreen wiper motion, from superficial to deep, from deep to superficial, and from medial to lateral. This was always in the same plane in order to make a single linear cut.

The bevel of the Abbocath acted as a knife on the weakened fascia, thus enabling a rapid and controlled advance of the cut.

During the perforations and the cutting movement, the assistant must hold the knee in extension and the ankle in forced dorsal flexion in order to create tension in the tendon and, therefore, make the release more effective.

The partial or total resection of the tendon will depend on the lengthening of the tendon that the surgeon wants to achieve for the correction of equinus. The enlargement can be controlled at all times with forced dorsal flexion.

**Fig. 4:**
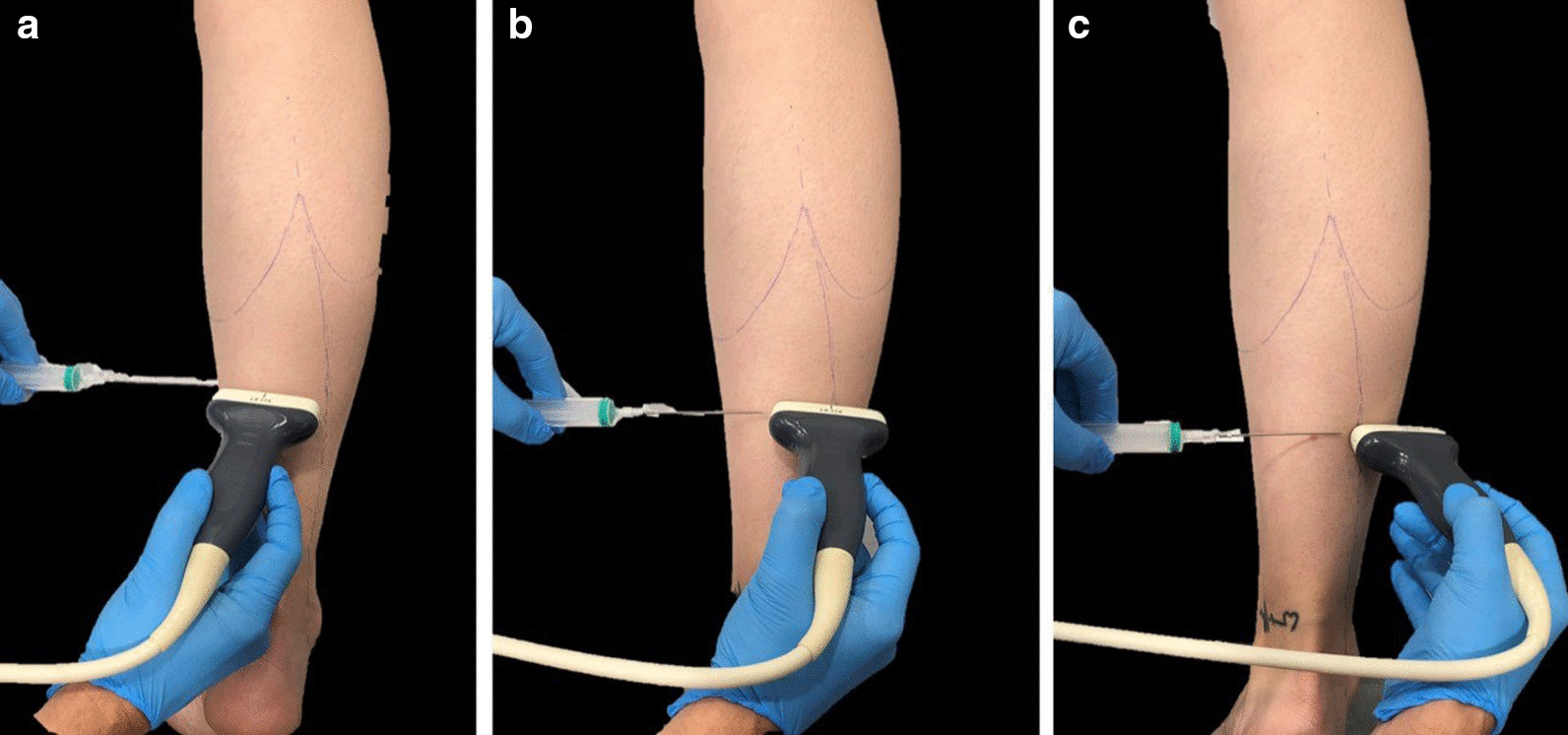
**a**–**c** The needle at 1, 2, or 3 different entry points from medial to lateral

The surgeon can perform partial or complete resection of the tendon, as described by Strayer [20]. Complete resection requires the surgeon to introduce the needle at 1, 2, or 3 different entry points from medial to lateral, as the resection advances. The surgeon must always maintain the same transverse ultrasound plane in order to achieve a continuous cutting line, thus preventing tendon fibers from remaining uncut and the needle from bending (Fig. 4a–c).

With the hook-knife technique, we advance the knife in a horizontal plane before turning it toward the tendon. Thus, the dome of the hook prevents the sural nerve from being transected, and the cutting edge points toward the tendon.

The point of greatest risk with the needle technique is recession of the tendon under the nerve and sural vein.

Therefore, serum is injected to separate the nerve from the tendon and enable the needle to be placed between them. The windscreen wiper movement should be performed exclusively from superficial to deep when the tip of the Abbocath is located underneath the nerve. For the rest of the tendon, it can be performed in both directions.

In any case, the nerve is not as rigid as the tendon, and the bevel does not cut a loose structure as easily as it does a rigid one. Consequently, it would be very difficult to damage the nerve.

Release is checked by palpating the needle and continuously observing the ultrasound image. The needle is moved from superficial to deep and vice versa and manually checked for absence of tension. In addition, the process can be visualized directly by ultrasound in both the transverse and the longitudinal planes.

At the end of the procedure, the needle can be withdrawn easily through the tendon, from the most posterior area of the soleus to the tendinous fascia, thus demonstrating that there are no fibers left to cut.

## Results

Secondary dissection of the area of recession confirmed complete release of the gastrocnemius Achilles tendon in all eight specimens. There was no damage to the sural nerve or vessels. Minimal damage to the underlying soleus muscle fibers was observed. We considered the technique to be sufficiently safe and effective for needle-based, ultrasound-guided recession of the gastrocnemius Achilles tendon.

**Fig. 5:**
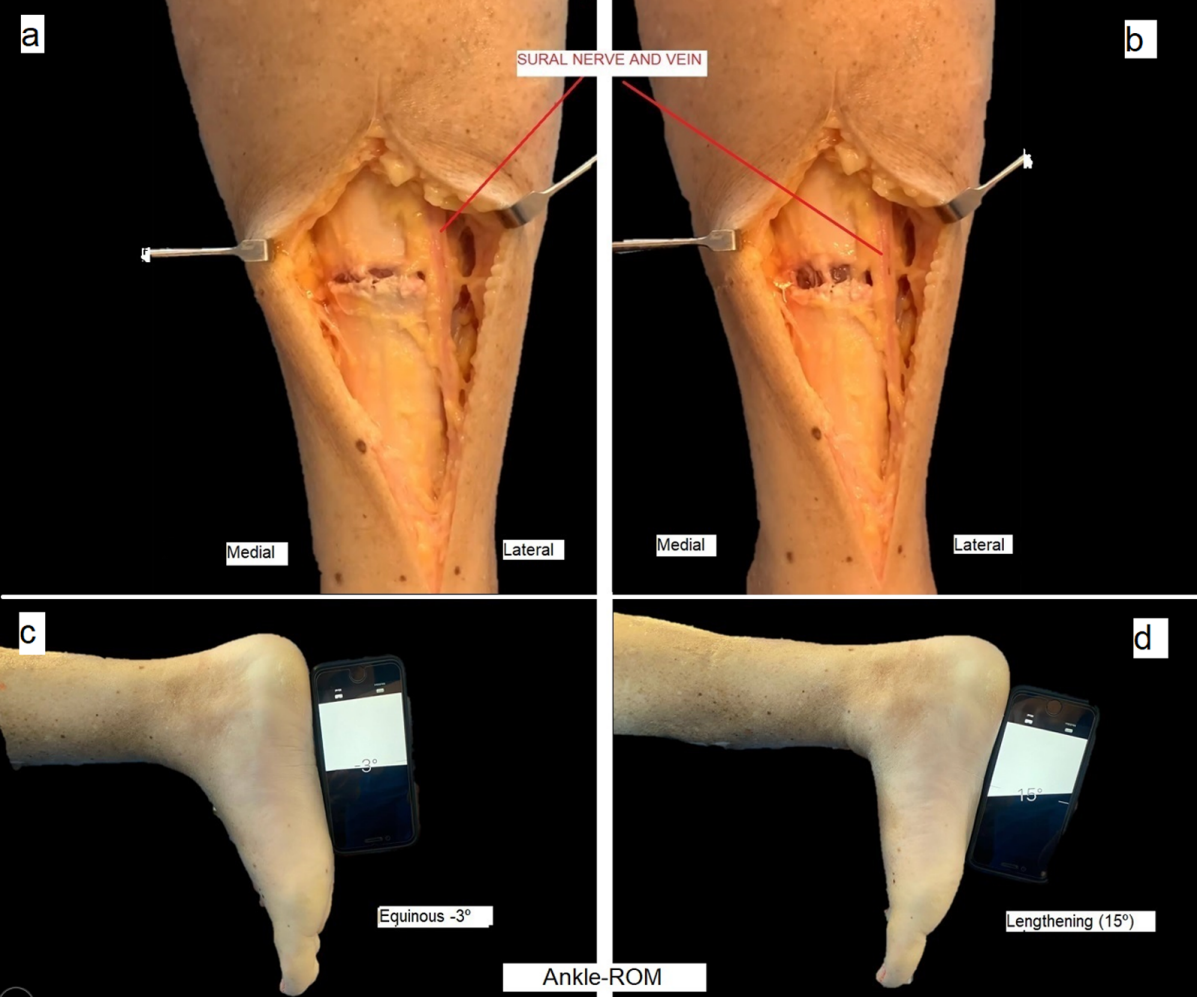
**a**, **b** Postsurgery Gap in Achilles tendon before and after forcing the ankle in dorsal flexion. **c**, **d** Ankle range-of-motion( Ankle-ROM) presurgery and postsurgery with the ankle in forced dorsal flexion

An average of 15° in dorsal flexion was obtained in all the specimens and at maximum tension in dorsal flexion. The gap in the tendon measured 11–17 mm (Fig. 5a–d).

The surgical procedure (total resection) took 9 (± 3) minutes.

## Discussion

The aim of the study was to evaluate the feasibility and safety of Strayer-type gastrocnemius tendon recession using a needle and guided by ultrasound.

The two least invasive surgical operations described to date are endoscopic surgery and ultrasound-guided surgery.

Endoscopic gastrocnemius recession is less invasive and may have advantages over open procedures in terms of enhanced visualization, smaller incisions, shorter operative time, fewer complications, and reduced morbidity [28, 34]. Endoscopic recession still requires epidural anesthesia, lower limb ischemia, and stitches. There is also a real possibility of damaging the sural nerve, which is not under continuous visualization, mainly at the first part of the portal [35]. An ultrasound-guided endoscopic procedure assisted to localize the sural nerve could diminish this complication.

Both ultrasound-guided and endoscopic surgery are performed using a hook-knife. This approach can be applied in the Ponseti technique for Achilles tendon lengthening in children in order to avoid neurovascular complications due to blind percutaneous tenotomy without direct visualization of the vessels or nerves [36, 37].

We reported on the effectiveness and safety of proximal and distal ultrasound-guided gastrocnemius release in 2016 and 2018 [11, 22]. Ultrasound-guided surgery improves on the disadvantages of endoscopic surgery, since it does not require ischemia and is performed with local anesthesia and sedation. Ultrasound-guided procedures enable continuous visualization of nerves and vessels, unlike endoscopic procedures, where the nerve must be identified after inserting the instruments and camera, thus placing it at risk during the initial stages.

The technique presented in this article is as effective as these approaches. In addition, it less traumatic, since the scalpel is replaced by a 16 G Abbocath.

The advantages of ultrasound-guided ultraminimally invasive surgical lengthening of the gastrocnemius include minimum risk of infection, minimum invasiveness (similar to that of an infiltration), no need for exsanguination, and local anesthesia. Moreover, the procedure is inexpensive and can be performed in the office under local anesthesia, without the need for an anesthesiologist or operating room.

The technique described in this article is based on previous approaches, namely, the ultrasound-guided release of the carpal tunnel, ultrasound-guided plantar fascia release, ultrasound-guided aponeurotomy, and interphalangeal joint capsular release for treatment of Dupuytren's disease. All these techniques can be performed in the office using a simple needle. Complications are minimal, recovery is rapid, and costs lower than procedures requiring an operating room [33, 38, 39].

Specific real-world studies are necessary to establish the feasibility of these theoretically complication-free advantages.

## Conclusion

Our study shows that needle-based, ultrasound-guided gastrocnemius lengthening is sufficiently safe, with no damage to major anatomical structures.

Ultrasound-guided needle-based gastrocnemius lengthening could be an alternative to traditional surgical treatment of gastrocnemius contracture.

## Data Availability

Can be requested from the corresponding author.
